# Study protocol: young carers and young adult carers in Switzerland

**DOI:** 10.1186/s12913-018-2981-5

**Published:** 2018-03-15

**Authors:** Agnes Leu, Corinna Jung, Marianne Frech, Joe Sempik, Urs Moser, Martin Verner, Saul Becker

**Affiliations:** 1 Kalaidos University of Applied Sciences, Careum Research, Pestalozzistrasse 7, CH-8032 Zurich, Switzerland; 2 Institut für Hausarztmedizin der Universität Zürich, UniversitätsSpital Zurich, Pestalozzistrasse 24, CH-8091 Zurich, Switzerland; 3 School of Social Policy, University of Birmingham, B15 2TT, Edgbaston, Birmingham UK; 4 Institut für Bildungsevaluation, Assoziiertes Institut der Universität Zürich, Wilfriedstrasse 15, 8032 Zurich, Switzerland; 5 University of Birmingham, Edgbaston, B15 2TT, Birmingham, UK

**Keywords:** Young Carers, Young Adult Carers, Switzerland

## Abstract

**Background:**

In Switzerland, the issue of young carers and young adult carers - young people under the age of 18 and 24 respectively, who take on significant or substantial caring tasks and levels of responsibility that would usually be associated with an adult - has not been researched before. The number of these younger carers is unknown, as is the extent and kind of their caring activities and the outcomes for their health, well-being, psycho-social development, education, transitions to adulthood, future employability and economic participation.

**Methods:**

The project is comprised of three stages:

1. A national Swiss-wide online survey to examine awareness of the issue of younger carers amongst professional populations in the education, health and social services sectors;
2. An online survey of 4800 Swiss pupils in schools using standardised instruments to identify the proportion and characteristics of pupils who are carers; and
3. Semi-structured interviews with 20 families comprising family members with care needs and younger carers, to consolidate and validate the other stages of the study; and to hear directly from care-dependent family members and younger carers about their experiences of the issues identified in the surveys and in previous published research.

**Discussion:**

The needs of younger carers and their ill and disabled family members in Switzerland have not been systematically investigated. This will be the first study in the country to investigate these issues and to develop evidence-based recommendations for policy and practice, drawing also on international research.

The present study therefore fills an important national and international research gap. It will collect important data on the awareness, extent, kind and impact of caring amongst children and young people in Switzerland, and cross-link these findings with robust evidence from other countries. The study will reveal (a) the extent of awareness of the issue of young carers amongst medical, social, health, educational, and other groups in Switzerland; (b) the proportion and number of young carers amongst a normative child population, and what these young carers ‘do’ in terms of their caring roles; and (c) direct accounts by families of their care-giving and receiving experiences.

## Background

Until the 1990s, the role that children and young people played in providing informal family care received virtually no academic or policy recognition. Since then, research into the area has substantially increased our understanding of these children and their lives and difficulties. Children who provide such care are generally referred to as «young carers» [1], although different countries use different terms. For example, the term «young caregiver» is used in the United States [2], and «children who are next of kin» is used in Norway and Sweden [3]. Although a number of definitions of these exist, most have the following common features: young carers are young people under the age of eighteen who take on significant or substantial caring tasks and levels of responsibility that would usually be associated with an adult. The person receiving care is often a parent but can be a sibling, grandparent, or another relative with a disability or a chronic illness, mental health problem or condition requiring care, support, or supervision [1]. It is important to note that the type of care provided by young carers extends beyond the basic household chores that many children and young people carry out. Research shows that young carers provide care in all areas where care is needed, including physical and emotional care (including intimate personal care), household management and taking care of siblings [4–6]. The reasons for providing care are complex and often related to the absence of other informally available networks, the lack of suitable formal care arrangements, familial preferences as well the natural family bond to the person in need [4–8].

More recently, Becker and Becker [9] broadened the research by including older children and young people and defining a “young adult carer”, as a person aged between 18 and 24, who provides care or support to another family member free of charge. Research has shown that these young people also face substantial difficulties in education and in employment [10–12]. Furthermore, according to international studies, young adult carers are often unemployed, or work part time in order to be able to carry on with their caring responsibilities. They may have no appropriate qualifications for employment because of the impact that their caring role has had on their education and training [13].

Research has highlighted the negative outcomes that caring has on children, for example, restricted social and educational opportunities. Much of this work has originated in the United Kingdom (see [14, 15]) and it has had an impact on policy and legal provision in that country, with specific legislation to protect young carers and a national network of hundreds of service programmes. Apart from a few other countries including Australia, Canada, New Zealand, Norway, Sweden, Germany, Austria, and France, most nations and governments have not engaged in identifying and supporting young carers, even though it is likely that between two and 4 % of all children (as a minimum estimate) take on caring roles within their families. This reflects a lack of awareness about children who care and the contributions that they make to informal family care, as well as a lack of engagement by academics and by policymakers.

By the end of 2011, Switzerland had a residential population of just under eight million people, with 2.1 million children, adolescents and young adults aged up to 24 years (1.6 million aged up to 19 years, 0.5 million aged 19 to 24 years). Therefore, if the findings from other countries can be extrapolated to Switzerland, there are at least 33,000 to 66,000 child and adolescent carers under 19; and an additional 22,000 to 26,000 young adult carers aged 19–24 living in Switzerland. These carers need to be identified so that they and their families receive proper support and the children and young people are do not (need to) provide excessive or inappropriate care-giving, or that which involve risky situations.

However, there is little awareness of children, adolescents and young adults under 24 involved in caring for family members in Switzerland. The numbers of these so called “young carers” and “young adult carers”, the sort and the extent of their tasks, as well as their impact are unknown. The needs and wishes of young people with a caring role and their families are also unexplored.

### The international situation

Studies from England, Australia, USA, Canada, Africa, Germany, Austria and Scandinavian countries have all shown that there are children, adolescents and young adults who regularly look after a chronically ill family member; hence this is a global phenomenon. Estimates of the number of child, adolescent and young adult carers differ between countries. In Great Britain, around 2.1% of children under 18 years are carers [9]); in Austria [8] and Australia [13] the figure is 3.6%; and in the US it is 3.2% [16].

Sweden and Norway are in the process of trying to identify and «count» the numbers of young carers in their countries using a methodology of self-disclosure.

The kind of support children provide for their families ranges from household tasks (cleaning, grocery shopping, cooking), helping siblings (looking after them, doing homework together, putting them to bed, taking them to nursery or to school), to support regarding their illness (physical and emotional support and personal hygiene care) [16, 17].

The amount of care carried out by children, adolescents and young adults is extensive; 14% of child and adolescent carers under 18 in Austria, for example, state that they are providing care for five to six hours per day [8]; in Great Britain the figure is 9% [9]. This shows the level of responsibility that these children have in their everyday life, many of whom started caring from an early age.

## Methods

### Study aims

Considering the findings from international research, it is likely that children and young adults in Switzerland also play a significant role in delivering familial support. The current study, therefore, explores the personal, familial and social situations of young carers and young adult carers. The study’s aims are to:

1. Learn more about awareness of the issue of young carers and young adult carers among health care, education, and social services professionals.
2. Estimate the number of children, adolescents and young adults who act as family carers. Such data are essential for the creation of appropriate and adequate support services.
3. Investigate the type and the intensity of children’s caring roles, their pathways into care-giving, the socio-economic and demographic circumstances of the young people and their families.
4. Examine the participation of children, adolescent and young adult carers in education, employment and social activities, and their plans for the future.
5. Make recommendations for supportive programmes for younger carers, based on existing structures but also involving new structures which are especially oriented to the target group. The pursued objectives are the perception and acceptance of child, adolescent and young adult carers as a social reality in Swiss society; the protection of children’s rights and their participation in all spheres of life which are relevant to their age and maturity; and, as a preventative measure, the welfare and promotion of the health of younger carers as well as support for the family as a whole.
6. Make young carers in Switzerland visible and increase the awareness of this group among professionals and the public. Good estimates of the prevalence of young carers in Switzerland, as provided by this project, and details of their support needs will show clearly that young carers do exist in the country and that they do require services and support. In the UK, for example, such research findings were instrumental in changing the law and policies towards protecting young carers. Additionally, research has shown that nationally-produced research is necessary for policymakers [18] to achieve change. Hence, by publication of the findings through a range of media we intend to make young carers ‘visible’ to policymakers, academics and health and social care professionals.

### Study design

The project comprises of three stages:

1. A national Swiss-wide online survey to examine awareness of the issue of younger carers among professional populations in the education, health and social services sectors;
2. An online survey of 4800 Swiss pupils in schools using standardised instruments to identify the proportion and characteristics of pupils who are carers; and
3. Semi-structured interviews with 20 families comprising family members with care needs and younger carers; to consolidate and validate the other stages of the study; and to hear directly from care-dependent family members and younger carers about their experiences of the issues identified in the online surveys and previous published research.

### Methodology

#### Work package 1: Online survey of professionals’ awareness of young carers and young adult carers

The online survey will examine the awareness of the issue of young carers and young adult carers among professional populations. The survey will target general practitioners (GPs), specialists (doctors dealing with somatic and psychiatric chronic diseases, specialists working with mental illness, drug-, and/or alcohol addiction, experts in end of life care), social workers, teachers, nurses, education policy makers and health policy makers in order to evaluate the level of awareness and knowledge about children and young adults who are carers in Switzerland. Professionals in the three areas of health care, education and social services in all language parts of Switzerland will be included in the survey.

The results will provide a baseline of the current understanding of the issue in Switzerland; whether, overall, there is good or poor awareness; and whether there is a better awareness among some groups but not others. From this information, it will be possible to map out what professionals in Switzerland know about the situation of this vulnerable group of young and young adult carers.

#### Sample and field approach

An online survey tool will be developed for the use with the different groups of professionals. The aim is to recruit 1000 health and social care professionals whose daily work includes contact with children. These respondents are, therefore, likely to meet children who could potentially have caring roles. The sample will include teachers, school nurses, medical practitioners who have a direct involvement in schools, and child social workers. A convenience sampling approach will be used. Participants will be recruited via third parties i.e. with the help of existing contacts in the professions, and through approaching persons in charge of health care, education and social services organisations.

#### Data analysis

The data will be collected using an online survey (Survey Monkey) and the link will be sent by email to potential respondents. The data will then be formatted and prepared for analysis by IBM SPSS Statistics (version 23). Descriptive and inferential statistics will be used as appropriate.

The analysis will examine the overall level of awareness of young carers by health and social care professionals; whether there are differences in awareness between professionals working in different areas (for example, education and health); whether age or the length of time in the profession has an influence on awareness; and whether there are any geographical differences in levels of awareness. The analysis will also show how many young carers the respondents encounter in their professional role and how this varies between the different professions.

### WP 2: Online survey to identify the number and characteristics of young carers in Switzerland

The second quantitative work package will estimate the number of children, adolescents and young adult carers in Switzerland and the extent of their caring roles. A large sample of children in schools will be surveyed to identify those that have caring roles and those that do not. Additionally, those with caring roles will be asked to identify their care-giving activities and responsibilities. From these data, estimates of the extent and type of caring among young people in Switzerland, as a country overall, will be calculated and compared with similar prevalence data from other countries. This will provide a measure of the extent to which Swiss youth are involved in family care and how this compares to other countries of a similar socio-economic status.

#### Sample and field approach

In the academic year 2013/2014 in Switzerland, there was, according to the survey of the Swiss Federal Statistical Office, 10,630 educational institutions in 26 cantons, including nursery schools, primary education institutions and different types of secondary educational institutions (public, privately-subsidised and privately non-subsidised institutions) [19]. The present study focuses on pupils and students enrolled in secondary educational institutions. Hence, the population of interest consists of 2367 institutions teaching approximately 630,000 pupils and students. Out of this population, 4800 pupils will be selected by means of a two-step sampling procedure. In the first step, a probability proportional to size (PPS) school sample will be drawn. Within sampled schools, a particular number of classes will be selected across available grades.

In order to ensure representativeness, the school frame will be stratified (explicit stratification) by language region (German, French, Italian) and school stage (first and second stage of secondary education). Within these explicit strata, schools will be sorted (implicit stratification) by canton, school type, school sponsorship and school size. School type refers to different performance-related study programs. While in most Cantons the first secondary school stage is divisible into three skill levels, the later secondary stage consists of six different programs, such as preparatory courses for the university entrance certificate, vocational education or diploma schools.

The total number of sampled schools will be allocated to the six explicit strata in a proportional manner. The exact number of schools and classes drawn per strata will be calculated after extensive analyses concerning school and class size variability.

The school-based survey will use self-completion questionnaires which have also been used in other countries (for example in the UK, Norway and Sweden), thus enabling cross-national comparisons to be made with a large Swiss sample. The questionnaires include the psychometric instrument MACA-YC18 (Multi-dimensional Assessment of Caring Activities 18 Item Scale, [20]) developed in England and used widely internationally, and a standard questionnaire of well-being, supplemented by other biographical questions. Because the questionnaires will be administrated by schools who have agreed to take part in the research (and which support its aims), we expect a large response rate.

The identification of young carers out of a greater collective of pupils is a challenge in terms of theoretical measurement and practical research because often they do not perceive themselves as carers. Families, in which children carry caring responsibility, often consciously avoid this kind of identification. The MACA is a proven tool which reveals caring among children who hitherto may not have regarded themselves as young carers.

The questionnaire has to be administered by schools following training, so that the majority of young carers, as well as those who do not have a caring role, feel motivated to complete the questionnaire. We will work closely with the selected schools to ensure they have full understanding of the purpose of the research and receive training in how to administer the questionnaires to their pupils. Schools will be provided with a DVD presentation about the research and the questionnaires, and they will also have access to a researcher who can be physically available on site to assist with the administration of the questionnaires if required.

#### Data analysis

The survey data will be collected by using an online system and then transferred to a *Microsoft Access* database that has been specifically developed for the research. The data will then be formatted and prepared for analysis by IBM SPSS Statistics (version 23) to produce descriptive statistics showing distributions of age, gender, location and other variables. In terms of inferential statistics, parametric and nonparametric methods will be used, as appropriate. The analysis will determine the prevalence of young carers within the sample and this will be extrapolated to the general population using complex sample analysis. The analysis will explore whether there is a difference in prevalence of young carers by gender and by geographical region; in what way it is influenced by age; and whether there is a difference in the extent of caring (as measured by the MACA-18) between genders and age groups. Additionally, the analysis will examine whether there is an association between the extent of caring (MACA-18) and the children’s wellbeing (using ‘Kidscreen-10’ [21],a measure of health-related quality of life which will be included in the survey).

### WP 3: Interviews with younger carers and their ill family members in Switzerland

This final part of the project enables us to test out our findings from the previous work packages through direct face to face interviews with young carers and the family members who receive care across Switzerland. Using a semi-structured interview schedule alongside psychometric instruments which measure the extent and nature of caring and the impacts of caring on children (MACA-YC18, PANOC [20]), we will be able to (a) hear directly from families about their caring experiences, interventions and support received, and to (b) validate the findings from other parts of the study. For example, young carers and their care-dependent family members’ accounts of the support they have received in education, health and social services will be examined in the context of the awareness of the issue of young carers among professionals in those fields (data collected in work package 1).

#### Sample and field approach

A sample of 20 families will be recruited where there is a care-dependent person with an illness, disability or mental health problems and at least one child or young adult in that family is a carer (purposive sampling). This will provide at least 20 interviews with care-dependent family members and 20 interviews with younger carers (*n* = 40). In some families, there may be more than one child who is a carer and this will increase the sample size. Interviews with care-dependent family members and children will be conducted separately. Families will be recruited through medical practices, health associations and social services and NGOs working with specific family types (e.g. MS Society, Parkinson’s disease Society, Palliative Associations, etc.).

#### Data analysis

Interviews with younger carers and care-dependent family members will be recorded and fully transcribed for thematic data analysis using a grounded theory approach [22]. Psychometric data will be collected using the MACA-YC18 ([20] as in work package 2) and the PANOC-YC20 (Positive and Negative Outcomes of Caring 20 Item Scale [20]). This will enable a ‘score’ to be determined for the level of caring among each of the children and a measure of their positive and negative adaptation to caring (PANOC). A standardised measure of health and well-being will be used with the family members.

## Discussion

The synthesis of these three work packages will make a major contribution to new knowledge and enable the research team to develop recommendations for evidence-based policy and practice to meet the needs of younger carers and their families in Switzerland. The aim is to enable practitioners to be better able to recognise and identify younger carers in whatever settings they may be; and to make policy and practice recommendations for interventions to promote the development, education, health, well-being and future employability of younger carers in Switzerland.

The situation of younger carers in Switzerland has not been researched before. The number of these younger carers is unknown, as is the extent and nature of their caring activities and the outcomes for their health, well-being, psycho-social development, education, transitions to adulthood and future employability and economic participation.

The needs of younger carers and their ill and disabled family members have not been systematically investigated. This will be the first study in Switzerland to investigate these issues and to develop evidence-based recommendations for policy and practice, drawing also on international research.

The present study therefore fills an important national and international research gap. It will be the first study to collect important data on the awareness, extent, type and impact of caring activities among children and young people in Switzerland, and to cross-link these findings with robust evidence from other countries. The study will reveal (a) the extent of awareness of the issue of young carers among medical, social, health, educational, and other groups in Switzerland; (b) the proportion and number of young carers in the normative child population, and what these young carers ‘do’ in terms of their caring roles; and (c) direct accounts by families of their care-giving and receiving experiences.

By publicising our method we hope that countries which as of yet have not conducted any studies into the lives and conditions of young carers and young adult carers will use the same instruments and protocols to carry out rigorous research to enable meaningful cross-national comparisons.

## Abbreviations

GP: general practitioner
MACA-YC18: Multi-dimensional Assessment of Caring Activities 18 Item Scale
PANOC-YC20: Positive and Negative Outcomes of Caring 20 Item Scale
